# Variability in detection and quantification of interferon β-1b–induced neutralizing antibodies

**DOI:** 10.1186/1742-2094-9-129

**Published:** 2012-06-15

**Authors:** Hans-Peter Hartung, Bernd Kieseier, Douglas S Goodin, Barry GW Arnason, Giancarlo Comi, Stuart Cook, Massimo Filippi, Douglas R Jeffery, Ludwig Kappos, Timon Bogumil, Brigitte Stemper, Rupert Sandbrink, Yukiko Nakada, Haruhiko Nakajima, Susanne Schwenke, Stephan Lehr, Jürgen Heubach, Christoph Pohl, Joachim Reischl

**Affiliations:** 1 Heinrich-Heine-Universität, Moorenstraße 5, Düsseldorf, 40225, Germany; 2 University of California, Rm. M794, San Francisco, CA, 94143-0114, USA; 3 Surgery Brain Research Institutes, 5812 S. Ellis Av., SBRI J209 (MC 2030), Chicago, IL, 60637, USA; 4 Scientific Institute and University Hospital San Raffaele, Via Olgettina 60, Milan, 20132, Italy; 5 UMD New Jersey Medical School, 65 Bergen Street - Suite 1435, Newark, NJ, 07101-1709, USA; 6 The MS Center at Advance Neurology, Cornerstone Healthcare, 152 Kinderton Way, Suite 101, Advance, NC, 27006, USA; 7 University Hospital, Petersgraben 4, Basel, CH-4031, Switzerland; 8 Bayer HealthCare Pharmaceuticals, P.O. Box 1000, Montville, NJ, 07045-1000, USA; 9 Bayer Pharma AG, Sellerstr 31, Berlin, 13342, Germany; 10 Mitsubishi Chemical Medience Corporation, 3-30-1, Shimura, Itabashi-ku, Tokyo, 174-8555, Japan; 11 University Hospital of Bonn, Sigmund-Freud-Str. 25, Bonn, 53127, Germany; 12 Department of Neurology, Heinrich-Heine-Universität, Düsseldorf, D-40225, Germany

**Keywords:** Multiple sclerosis, Clinical trials randomized controlled, IFNB-1b, Interferon beta, Neutralizing antibodies, Round robin

## Abstract

**Background:**

Interferon-beta (IFNB) therapy for multiple sclerosis can lead to the induction of neutralizing antibodies (NAbs) against IFNB. Various methods are used for detection and quantification of NAbs.

**Methods:**

Blood samples from 125 IFNB-1b–treated patients, which were tested NAb negative or NAb positive after conclusion of a clinical study, were retested three years after first being assessed in four different laboratories that offer routine NAb testing to practicing neurologists. The myxovirus protein A (MxA) induction assay, the cytopathic effect (CPE) assay (two laboratories), or the luciferase assay were used. Intra- and inter-laboratory agreement between assays with respect to NAb detection and NAb titer quantification were evaluated.

**Results:**

High agreement for NAb detection (kappa coefficient, 0.86) and for titer levels was observed for the intra-laboratory comparison in the laboratory using the MxA induction assay performed three years ago and now. A similarly high agreement for NAb detection (kappa coefficient, 0.87) and for titer quantification was noted for the MxA assay of this laboratory with one of two laboratories using the CPE assay. All other inter-laboratory comparisons showed kappa values between 0.57 and 0.68 and remarkable differences in individual titer levels.

**Conclusions:**

There are considerable differences in the detection and quantification of IFNB-induced NAbs among laboratories offering NAb testing for clinical practice using different assay methods. It is important that these differences are considered when interpreting NAb results for clinical decision-making and when developing general recommendations for potentially clinically meaningful NAb titer levels.

## Introduction

Up to 40% of people with multiple sclerosis (MS) treated with interferon-β (IFNB) develop IFNB neutralizing antibodies (NAbs) [1]. Anti-IFNB NAbs have been associated with reduced therapeutic efficacy [2] exemplified by an increased annualized relapse rate and increased disease activity on brain magnetic resonance imaging. Furthermore, *in-vitro* studies have demonstrated that NAbs can lead to alterations in the transcription rate of MS-relevant genes [3,4]. In contrast, other studies have indicated that the relapse rate is not significantly different between NAb-negative and NAb-positive patients [2]. Generally, the frequency of NAbs against IFNB diminishes over time, and especially patients who develop NAbs to IFNB-1b (Betaferon®, Chiron Corporation, Emeryville, CA, USA) often revert to NAb-negative status upon subsequent testing [5-9]. High NAb titers appear to be more persistent and thus may have a greater impact on the efficacy of IFNB-1b [2,10,11].

Part of the inconsistent findings with regard to the clinical relevance of NAbs might result from the fact that various methods are used for evaluating NAbs in MS patients treated with IFNB and that IFNB-1a and -1b–treated patients are assessed jointly in some studies on NAbs. The objective of this study was to compare NAb detection and quantification of NAb titers in laboratories offering NAb testing for treatment decision making in clinical routine. These laboratories use different assay methods, that is, the myxovirus protein A (MxA) induction assay and the cytopathic effect (CPE) assay [1,2,12].

## Methods

### Study design

Blood samples obtained in the Betaferon Efficacy Yielding Outcomes of a New Dose (BEYOND) study were used. The BEYOND study was a randomized, parallel group, Phase 3 study conducted across 198 centers in 26 countries worldwide [13]. In total, 2,244 patients with relapsing-remitting MS were enrolled and randomly assigned in a ratio of 2:2:1 to receive one of two doses of IFNB-1b (either 250 μg or 500 μg) subcutaneously every other day or 20 mg glatiramer acetate subcutaneously every day. Serum samples for NAb testing were collected at baseline and then every six months under treatment. At the end of the study, these samples were tested for NAb positivity and for NAb titer quantification with an MxA induction assay. A sample was considered “NAb positive” with a titer of at least 20 units (lower limit of quantification, LLOQ) using this assay. If no quantifiable NAb titer is detectable, the respective sample was considered “NAb negative.” Comprehensive details of the measurement, quantification and NAb titers in the BEYOND study have been reported previously [14]. The Institutional Review Boards of all participating centres approved the study protocol and all patients gave written informed consent before trial entry.

The present study used serum samples of the BEYOND study. Of serum samples obtained 1.5 years after the start of IFNB-1b 250 μg treatment, 125 were selected for the intra- and inter-laboratory comparison based on the original test results from Laboratory A (A(I)). Sample selection was not representative of the NAb status distribution nor of NAb titers observed in the BEYOND trial, but optimized for dense and steady coverage of the entire NAb titer range (n = 82) while including enough NAb-negative samples (n = 43). The samples had been stored at −20° and thawed and frozen once during aliquoting. Three years after the original NAb analysis, sample aliquots were reanalyzed at Laboratory A using the same MxA induction assay (A[II]). In addition, the aliquots were tested in three other laboratories using the CPE bioassay (Laboratories B, LLOQ = 8, and C, LLOQ = 20) and the luciferase bioassay in Laboratory D (LLOQ = 20). There was no upper limit of quantification for Laboratories A and B, but it was 640 for the CPE assay performed at Laboratory C and 1,202 for the luciferase assay of Laboratory D. The principles of NAb testing using these three bioassays have been published previously [15-19].

All of the laboratories that assayed the samples for neutralizing antibody activity in this study offer neutralizing antibody testing in clinical practice, but it was agreed that they would remain anonymous when reporting the results of this study. The ability of neutralizing antibodies to block the biological activity of IFNB, which is dependent on the molecule binding to its receptor, is measured in neutralization assays. In the MxA assay, serum samples were mixed with IFNB-1b and incubated with A549 cells (human embryonic lung cells) [13]. Cell lysates were then tested for MxA protein using ELISA. The neutralizing titer was the reciprocal serum dilution that reduced the MxA-inducing activity 10-fold. At the end of the BEYOND study and after storage for three years, the samples were tested by Rentschler, Laupheim, Germany.

In the luciferase assay, HL116 cells stably transfected with a luciferase reporter gene cassette were used, as described before [18]. Briefly, a transcellular signaling mechanism is activated when the IFNB molecule binds to its receptor, which activates the IFN-stimulated response element. This translocates to the nucleus where it causes the transcription of the luciferase gene; the resulting luminescence signal was read by a conventional reader. The amount of luciferase produced in response to a known quantity is predictable, but this response is blocked by neutralizing antibody. IFNB-1a was used in this assay.

A number of CPE assays have been developed using a variety of cell lines and viruses to determine the titer of an IFN sample. The addition of neutralizing antibodies to the interferon allows the titer of neutralizing antibody to be quantified. Many of these assays provide a rapid, simple and sensitive assay. The CPE assay reported under laboratory B was performed by the Mitsubishi Chemical Medience Corporation (Tokyo, Japan) using FL cells and Sindbis virus. IFNB-1b was used in this assay. The IFN used in the CPE assay performed by laboratory C was not specified. No details about the assay were provided and the laboratory did not agree to disclose its identity for this publication.

### Statistical analyses

Patient samples with NAb titers below the LLOQ are defined as being NAb negative, and samples with quantifiable NAb titers as being NAb positive. The agreement between the different bioassays regarding NAb negative versus NAb positive status was assessed using the kappa coefficient. The kappa coefficient is commonly used in studies that measure agreement between two or more observations, since it accounts for the fact that observers will occasionally agree purely by chance [20]. A kappa of 0 indicates agreement by chance, whereas a kappa of 1 indicates perfect agreement [20]. Titer values were compared graphically by means of scatter plots.

## Results

### Distribution of NAb titers reported by different laboratories

**Figure 1:**
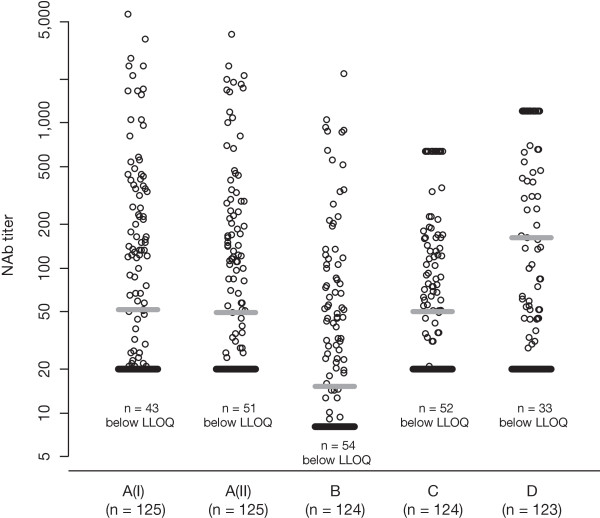
**Scatter plot of reported titer levels.** The number of valid results out of the 125 provided samples is given below each laboratory identifier. The gray bar indicates the median value, the black bar indicates the lower limit of quantification (LLOQ). Laboratories C and D had an upper limit of quantification, that is, values are censored. NAb = neutralizing antibody.

The titer levels reported by the different laboratories are shown in Figure 1. The median values were comparable in the original analysis (52) and in the reanalysis after three years (49) performed at Laboratory A, and in the analysis performed at Laboratory C (50). Median levels were lower in Laboratory B (15.3) and higher in Laboratory D (162). The maximum titers observed were in the same order of magnitude in Laboratories A (5,646) and B (2,180), but censored in the analysis at Laboratories C (640; n = 18 samples) and D (1,202; n = 44 samples). The percentage of samples below the laboratory-specific LLOQs ranged from 27% (Laboratory D) to 44% (Laboratory B).

### Intra- and inter-laboratory comparison of NAb titer values

**Figure 2:**
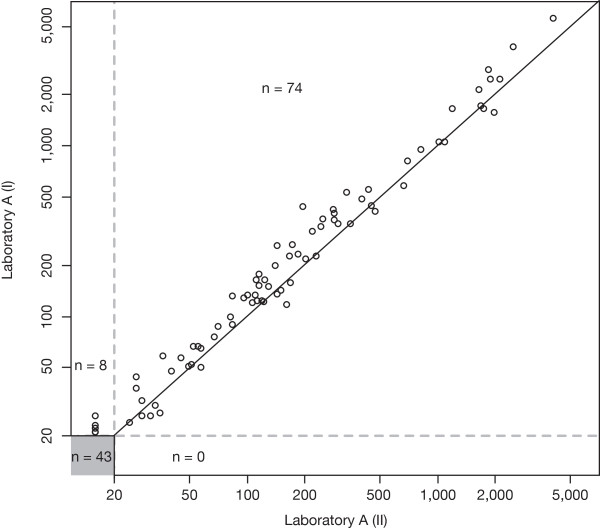
**Intra-laboratory comparison of NAb status and NAb titers at Laboratory A.** The titer value of a sample measured in Laboratory A (II) three years after the end of the study (x-axis) is plotted against the value of a sample measured in Laboratory A (I) shortly after the end of the study (y-axis). A total of 43 samples tested negative in both laboratory runs. The solid line indicates equal titer levels in both laboratories the dashed lines indicate the lower limits of quantification.

For the intra-laboratory comparison we assessed the agreement between the repeat NAb titer measurements at Laboratory A (Figure 2). All 43 samples with NAb titers below the LLOQ in the original analysis were confirmed in the reanalysis three years later. In addition, eight samples with low NAb titers in the original analysis yielded NAb titers below the LLOQ in the reanalysis. For the 74 samples with titer levels above the LLOQ in both laboratory runs, there was a high level of agreement across the entire range of titer levels.

**Figure 3:**
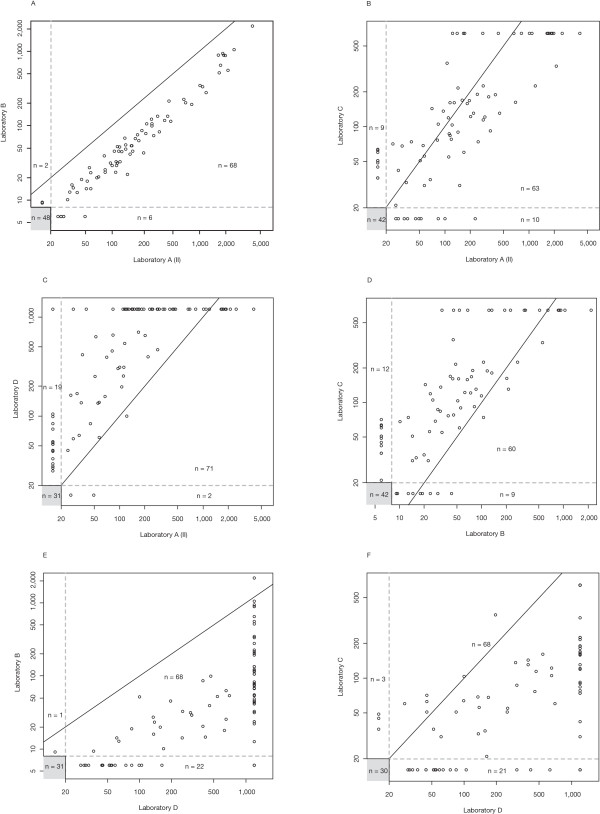
**Inter-laboratory comparison of neutralizing antibody (NAb) status and NAb titers.** The number of samples yielding values below the lower limit of quantification (LLOQ) in both laboratories is given in the gray boxes. The solid line indicates equal titer levels in both laboratories. Dashed lines indicate the LLOQs of the respective laboratories.

Inter-laboratory comparisons are shown in Figure 3 in a similar fashion. Figure 3a demonstrates that NAb titer levels resulting from Laboratory B were systematically lower than values determined at Laboratory A. The NAb titer values, however, were strongly correlated, despite the different NAb assay technologies applied (MxA induction assay at Laboratory A vs. CPE assay at Laboratory B). The correlation of NAb titers was much weaker when comparing the results from Laboratories B and C, which both applied the CPE assay principle (Figure 3d). Despite similar median NAb titer values in Laboratories A and C, the titer correlation was weak as well (Figure 3b). In comparison with all other laboratories, Laboratory D reported a relatively large number of samples with titer values above the LLOQ (Figure 3c, e and f). For samples with quantifiable NAb titers in comparisons involving Laboratory D, titer values tended to be higher in Laboratory D, and the correlations were weak.

### Consistency of NAb test results across laboratories

The agreement regarding the NAb status result (positive vs. negative) within Laboratory A and between laboratories is provided as kappa values in Table 1. The retest results at laboratory A reached a kappa value of 0.86, indicating an almost perfect agreement. This kappa value was even slightly exceeded for the NAb status comparisons in Laboratory A and Laboratory B, with a calculated kappa value of 0.87. The kappa values for the other inter-laboratory comparisons ranged from 0.57 to 0.68.

**Table 1:** Intra- and inter-laboratory agreement of the NAb testing in terms of NAb positive versus NAb negative status

**Laboratory**	**A(I)**	**B**	**C**	**D**
**A(II)**	0.86	0.87	0.68	0.63
**B**			0.65	0.60
**C**				0.57

Kappa values are shown for the individual laboratory comparisons. A (I): original analysis in Laboratory A (MxA induction assay); A(II) re-analysis of samples in Laboratory A (MxA induction assay); B, Laboratory B (CPE assay); C, Laboratory C (CPE assay); D, Laboratory D (luciferase assay).

**Figure 4:**
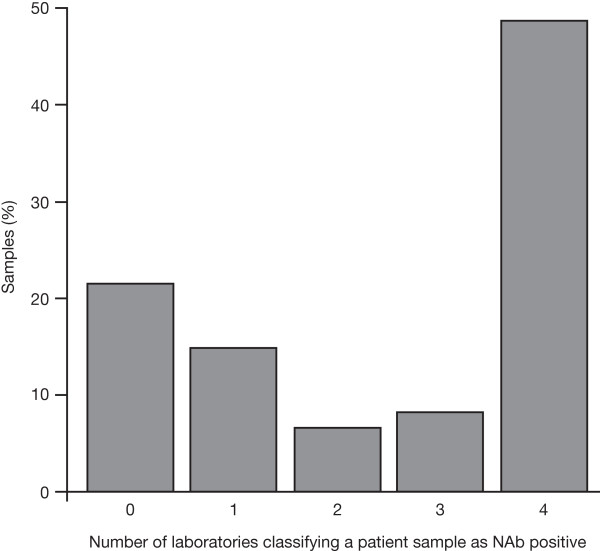
**Consistency of neutralizing antibody (NAb) status classification across Laboratories A (II), B, C and D.** The numbers on the x-axis indicate the numbers of laboratories that report a specific sample as being NAb positive. 0 = no laboratory reported a titer value above the lower limit of quantification (LLOQ); 1, 2, 3 = one, two or three laboratories out of four reported a quantifiable NAb titer; 4 = all four laboratories measured a NAb titer value above the LLOQ. The y-axis provides the percentage of samples in each category. This analysis involved the 121 patient samples with valid results from all four laboratories.

Special attention was paid to the question of whether a patient was consistently classified as being NAb positive or NAb negative by the four different laboratories, as this may have implications for further treatment. Figure 4 shows that, from the samples selected for this laboratory comparison, 22% were consistently reported as NAb negative and 48% were consistently reported as NAb positive. However, for about one-third of the samples (30%), the four laboratories involved reported inconsistent results regarding a patient´s NAb status.

## Discussion

Recently, an international panel of MS experts on NAbs to IFNB therapy convened under the auspices of the Neutralizing Antibodies on Interferon beta in Multiple Sclerosis consortium (NAbinMS: a collaborative project funded by the European Union) and issued recommendations for clinical use of data on NAb to IFNB therapy [21]. In this position paper, the use of the MxA induction assay is suggested to test neutralizing activity to IFNB therapy and a switch to a non-IFNB therapy is recommended in cases of sustained high-titer NAb positivity and/or lack of IFNB bioactivity. However, in clinical practice, other assays are also employed to detect and quantify the level of neutralizing activity to IFNB treatment, such as the CPE and the luciferase assay. These assays differ not only in their sensitivity and specificity but also in their performance in various laboratories resulting in differences with respect to NAb detection and titer quantification [22]. Furthermore, difficulties in standardizing these assays have considerably hindered inter-laboratory comparison of NAb data [23].

Here, we assessed the variability and agreement of NAb testing results between different methods and laboratories offering routine NAb testing to detect and quantify neutralizing activity to IFNB treatment in clinical practice. There was a high intra-laboratory agreement for NAb detection and quantification with the MxA induction assay that was used in the BEYOND trial. This finding reveals a robust stability of the neutralizing activity in patient sera stored for several years and a good retest reliability of the MxA induction assay that was used. With respect to the inter-laboratory comparisons, there remained high agreement of NAb detection and quantification between the MxA induction assay and one laboratory using a CPE assay, with just a systematic difference in titers. This contrasted with only moderate agreement between the MxA assay and the CPE assay used by a second laboratory and to the modest agreement between the NAb findings of the two CPE laboratories. Also, the agreement between the MxA assay and both CPE assays with a luciferase assay was only moderate. Of note, when comparing individual titer levels, substantial differences were recorded between laboratories: we frequently observed that high-titer NAb positivity was measured by one lab in a sample that was found to contain low-titer NAb positivity by another lab. Occasionally, even high-titer NAb positivity was reported for samples that were tested NAb negative by another laboratory using a different method.

The focus of this work was to investigate the differences in NAb testing and quantification between laboratories offering NAb services in clinical practice. More research is warranted to better understand the observed discrepancies. This research could constitute the starting point for further standardization of NAb testing. For instance, higher titers have been observed when IFNB-1a vs. IFNB-1b was used as the antigen to test neutralizing activity, a finding that is in line with the somewhat higher titers of our laboratory D using IFNB-1a in a Luciferase assay [24]. In light of our findings, results of NAb testing obtained in different assays/laboratories must be interpreted or compared with caution. This is of particular importance when titer thresholds are considered for clinical decision-making as there are both systematic shifts and potentially high variability between test methods and among laboratories. Because high-titer NAb positivity may be regarded as a sufficient reason to stop IFNB and switch to a non-IFNB product even if patients are doing well [21], a false-positive NAb titer might have disadvantageous therapeutic consequences. Therefore, our findings underscore the need for global standardization efforts whenever complex indirect biological assays are to be used. They also support the recommendation of NAb experts to supplement testing for NAbs with more direct measurements of IFNB-induced biological activity in patients undergoing IFNB treatment (such as the measurement of MxA induction following an IFNB injection) [21]. Yet, the predictive value of measuring IFNB activity needs still to be established in a well-designed prospective trial [25].

## Conclusion

There are differences in the detection and quantification of IFNB-1b-induced NAbs between different laboratories and the assay methods used in clinical practice. It is important that these differences are considered not only when interpreting NAb results but also when developing general recommendations for potentially clinically relevant NAb titer levels.

## Abbreviations

CPE, cytopathic effect; IFNB, interferon-β; LLOQ, lower limit of quantification; MS, multiple sclerosis; MxA, myxovirus protein A; NAbs, neutralizing antibodies.

## Competing interests

The BEYOND study and NAb testing performed for this manuscript was funded by Bayer Pharma AG.

Dr. Hartung has received personal compensation from Biogen Idec, Teva, sanofi-aventis, Novartis Pharma, Merck Serono and Bayer Schering Pharma AG for speaking and consulting services. The MS Center at the Department of Neurology Heinrich-Heine-University is in part supported by the Walter-and-Ilse-Rose Stiftung.

Dr. Kieseier has received honoraria for lecturing, travel expenses for attending meetings and financial support for research from Baxter, Bayer Schering, Biogen Idec, Medac, Merck Serono, Novartis, Roche, sanofi-aventis, Talecris and Teva Neuroscience, Inc.

Dr. Goodin has participated (or is currently participating) in several industry-sponsored clinical trials in multiple sclerosis. The sponsoring pharmaceutical companies for these trials have included (or do include) Ares-Serono, Merck-Serono, Novartis, Berlex Laboratories, Bayer- Schering Healthcare, Biogen-Idec, Schering AG and Teva Neuroscience, Inc. He has also lectured at both medical conferences and in public on various aspects of the epidemiology, diagnosis and management of multiple sclerosis. In many cases, these talks have been sponsored directly or indirectly by one or another of the above-listed companies. He has also served as a temporary *ad hoc* consultant to several of these organizations on several occasions.

Dr. Arnason has served as a consultant within the past year to Bayer Schering Healthare, sanofi-aventis, Questcor, Inc. and Acorda, Inc. He has participated in a clinical trial sponsored by Acorda and has received research grant support from Questcor.

Dr. Comi has received personal compensation for speaking and consultancy activities from Bayer Schering, Serono Symposia International Foundation, Merck Serono International, sanofi-aventis, Novartis, Biogen Dompè and Teva Pharmaceutical Industries, Ltd. in the past two years.

Dr. Cook has received personal compensation for consultations from advisory boards or lectures from Bayer Healthcare, Merck Serono, Teva Pharmaceutical Industries Ltd, Biogen Idec, sanofi-aventis and Actinobacter.

Dr. Filippi serves on scientific advisory boards for Teva Pharmaceutical Industries, Ltd. and Genmab A/S; has received funding for travel from Bayer Schering Pharma, Biogen-Dompè, Genmab A/S, Merck Serono and Teva Pharmaceutical Industries, Ltd.; serves on editorial boards of the American Journal of Neuroradiology, BMC Musculoskeletal Disorders, Clinical Neurology and Neurosurgery, Erciyes Medical Journal, Journal of Alzheimer’s Disease, Journal of Neuroimaging, Journal of Neurovirology, Magnetic Resonance Imaging, Multiple Sclerosis and Neurological Sciences; serves as a consultant to Bayer Schering Pharma, Biogen-Dompè, Genmab A/S, Merck Serono and Teva Pharmaceutical Industries Ltd.; serves on speakers’ bureaus for Bayer Schering Pharma, Biogen-Dompè, Genmab A/S, Merck Serono and Teva Pharmaceutical Industries Ltd.; and receives research support from Bayer Schering Pharma, Biogen-Dompè, Genmab A/S, Merck Serono, Teva Pharmaceutical Industries Ltd. and Fondazione Italiana Sclerosi Multipla.

Dr. Jeffery has received honoraria and consulting fees from Berlex, Serono, Teva Pharmaceutical Industries Ltd, Glaxo and Pfizer.

Dr. Kappos has received financial support for research activities from Abbott, Bayer, Bayer HealthCare Pharmaceuticals, Bayer Schering Pharma, Bayhill, Biogen Idec, Centocor, Eisai, Elan, Genzyme, Merck Serono, Neurocrine, Novartis, sanofi-aventis, Roche, Teva, UCB and Wyeth.

Dr. Bogumil is a salaried employee of Bayer HealthCare Pharmaceuticals, Montville, NJ.

Dr. Stemper, Dr. Sandbrink, Dr. Schwenke, and Heubach are salaried employees of Bayer Pharma AG, Berlin, Germany.

Dr. Lehr was a salaried employee of Bayer Pharma AG, Berlin, Germany.

Yukiko Nakada and Haruhiko Nakajima are salaried employees of Mitsubishi Chemical Medience Corporation.

Dr. Pohl and Dr. Reischl are salaried employees of Bayer Pharma AG, Berlin, Germany.

## Authors’ contributions

HPH has been actively involved in the conception, design and drafting the protocol of the BEYOND study and the reported biomarker analysis. He reviewed the statistical analysis, contributed to interpretation of the results, drafted the first version of this manuscript, actively contributed to further development of the manuscript and approved its final version. DSG, BGWA, GC, SC, MF, DRJ and LK were actively involved in the conception, design and drafting the protocol of the BEYOND study. They reviewed the statistical analysis of the reported biomarker data, contributed to data interpretation, gave critical revision for important intellectual content of the submitted manuscript, and approved its final version. SL and SS were responsible for the conception, design and biometrical analyses reported in this manuscript, have created the statistical analysis plan, interpreted the results, have actively contributed to writing and reviewing of the submitted manuscript, and have approved its final version. CP has been actively involved in supervising the BEYOND study and in setting up the protocol of the reported biomarker analysis, reviewed the statistical analysis, interpreted the results and has actively contributed to the writing and reviewing of the submitted manuscript. He approved the final version of the manuscript. TB and RS were actively involved in the conception, design and drafting the protocol and in supervising the BEYOND study. They reviewed the statistical analysis of the reported biomarker data, contributed to data interpretation, gave critical revision for important intellectual content of the submitted manuscript and approved its final version. BK, JFH and BS actively contributed to the conception and design of the reported biomarker analysis, reviewed the statistical analysis, interpreted the results, actively contributed to the writing of the submitted manuscript and approved its final version. YN and HN were responsible for the CPE assay in the Mitsubishi Chemical Medience Corporation and approved the final version of the submitted manuscript. JR actively contributed to the conception and design of the reported biomarker analysis, reviewed the statistical analysis, interpreted the results, actively contributed to the writing of the submitted manuscript and approved its final version. He coordinated and supervised the NAb laboratory comparison study. All authors read and approved the final manuscript.
